# Supplementary material for: Magnon-polaron control in a surface magnetoacoustic wave resonator
Source: Nat Commun. 2025 Nov 18;16:10116. doi: 10.1038/s41467-025-66301-x (PMC12627481; doi:10.1038/s41467-025-66301-x)
Supplement: Supplementary file 1 — Supplementary Information [file 41467_2025_66301_MOESM1_ESM.pdf]

Supplementary Information: Magnon-polaron control in a surface magnetoacoustic wave resonator

Kevin Künstle,[1,*] Yannik Kunz,[1] Tarek Moussa,[1] Katharina Lasinger,[1,2] Kei Yamamoto,[3] Philipp Pirro,[1] John F. Gregg,[2] Akashdeep Kamra,[1] and Mathias Weiler[1]

[1] *Fachbereich Physik and Landesforschungszentrum OPTIMAS, Rheinland-Pfälzische Technische Universität Kaiserslautern-Landau, 67663 Kaiserslautern, Germany*

[2] *Clarendon Laboratory, Department of Physics, University of Oxford, Parks Road, Oxford, OX1 3PU, United Kingdom*

[3] *Advanced Science Research Center, Japan Atomic Energy Agency, Tokai 319-1195, Japan*

Supplementary Note 1: Resonator characteristics

The one-port surface acoustic wave (SAW) resonator employed in this study features a reflection-mitigating double-electrode interdigital transducer (IDT) centrally positioned between two Bragg mirrors, which function as reflective gratings for the acoustic waves. The double-electrode geometry of the IDT is implemented to suppress internal acoustic reflections, which can interfere with the establishment of a coherent standing wave within the planar resonator cavity. Each Bragg mirror comprises $N_\mathrm{g} = 100$ reflector stripes with a uniform finger width and spacing of 500 nm. The IDT exhibits an effective finger number N_IDT of 41, where each effective finger consists of two spatially separated fingers (250 nm width and spacing) connected to the same electrical contact. This geometry facilitates the excitation and resonant enhancement of a SAW with a wavelength of 2 µm.

Building on Reference [1] and our previous work on the YIG/ZnO heterostructure [2], we selected a ZnO thickness to wavelength ratio of $h_\mathrm{ZnO}/\lambda \approx 0.5$ to ensure sufficient electromachanical coupling.

A: SAW Wavelength measurement using Brillouin Light scattering

The excited wavelength can directly be accessed using microfocussed Brillouin light scattering (μBLS) spectroscopy [3]. The μBLS signal intensity (I_BLS) is proportional to the time averaged absolute value of the out-of-plane displacement $|u_\mathrm{z}|$ [4]:

$$I_\mathrm{BLS}(x) \propto \int_{T_0} |u_\mathrm{z}|^2 \,\mathrm{d}t \propto \sin^2(kx) \; . \tag{1}$$

Assuming a simplified standing wave $u_\mathrm{z}(x,t) = u_{\mathrm{z},0}\sin(kx)\sin(\omega t)$, the intensity is proportional to $\sin^2(kx)$. Here, $u_{\mathrm{z},0}$ denotes the amplitude, k the wavenumber and ω the angular frequency. To determine the wave length of the excited standing wave, the μBLS laser spot was positioned between the mirror and the IDT. A 25 μm long linescan with a stepsize of 130 nm between mirror and IDT was conducted (x-direction). A microwave frequency exciting the central high-Q mode of the resonator was applied and the resulting data is presented in Supplementary Fig. 1.

* kuenstle@rptu.de

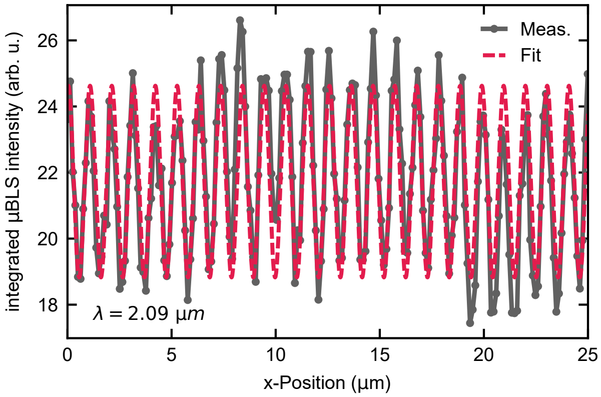

Supplementary Fig. 1: Measured integrated μBLS intensity over x-Position. Expected $\sin^2$ behavior is observed, from which a wavelength of $\lambda = 2.09\,\mu\mathrm{m}$ is determined.

By fitting the data to a function of the form $I(x) = A\sin^2(kx + \theta) + B$, the wave length was determined to be $\lambda = 2.09\,\mu\mathrm{m}$, which agrees well with the expected wavelength of $2\,\mu\mathrm{m}$ theoretically supported by the resonator's geometry. The observed variations in peak height can be attributed to several factors, including changes in reflection depending on the laser spot position on the ZnO surface. Additionally, the laser spot has a diameter of approximately 300 nm, depending on the focus position, and follows a Gaussian profile. This implies that the measured intensity is averaged over a region approximately one-sixth of the wavelength. Consequently, the intensity does not completely drop to zero, resulting in a constant offset.

B: SAW group velocity measurement using Brillouin Light scattering

To determine the group velocity (v_{gSAW}) of the excited surface acoustic wave, a time-resolved μBLS measurement was performed at a fixed frequency of 1.4 GHz. In this experiment, a short SAW pulse was generated at a specific time using an IDT distinct from the resonator structures. As the μBLS laser spot was incrementally positioned at increasing distances from this excitation IDT, the arrival time of the detected SAW pulse exhibited a corresponding shift due to the increased propagation path. The temporal profile of the detected SAW pulses was analyzed by fitting a sigmoidal Boltzmann function:

$$I_{\mathrm{BLS}}(t) = C + \frac{A - C}{1 + e^{-\frac{t - t_0}{B}}}\ , \tag{2}$$

where the time delay parameter t_0, representing the arrival time of the pulse leading edge at the laser spot, was extracted for each spatial position. Subsequently, a linear fit was applied to the extracted t_0 values as a function of the distance between the excitation IDT and the laser spot. The slope of this linear fit directly yields the group velocity of the SAW. A detailed description of this procedure can be found in Supplementary Ref. [2]. The group velocity determined through this method was found to be $v_{\mathrm{BLS}} = (2382.09 \pm 2.57)\,\mathrm{m\,s^{-1}}$. The experimental data illustrating this measurement is presented in Supplementary Fig. 2, which displays the integrated μBLS intensity of the measured SAW pulses as a function of both the distance from the sending IDT to the laser spot and time. The dashed red line overlaid on the figure represents the linear fit applied to the inflection points of the Boltzmann fits (Supplementary Eq. (2)) for each spatial position.

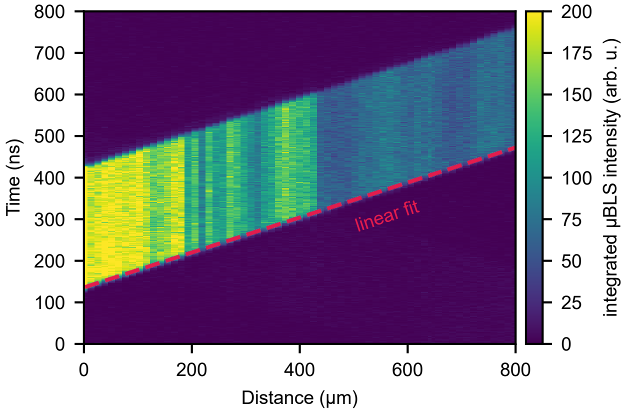

Supplementary Fig. 2: Integrated μBLS intensity in dependence of time and laser spot distance from the sending IDT. A SAW pulse is excited and detected at the position of the laser spot. By fitting the pulse flanks, the group velocity of the excited SAW was determined.

C: Evaluated resonator properties

From the electrical measurement presented in the main text [Fig. 2d)], combined with the μBLS measurements shown above, further resonator properties can be accessed. Having three high-Q modes within the first resonator stopband, which represent well-defined cavity resonances, we can determine the free spectral range (FSR) of these resonances. Knowing the geometric parameters of the mirror electrode width $a = 500\,\mathrm{nm}$ and the distance between mirrors $d = 302\,\mathrm{\mu m}$, the reflection per single electrode in the mirror (r_s) is calculated via [5]:

$$r_\mathrm{s} = \frac{2a}{\frac{v_\mathrm{gSAW}}{FSR} - d} = 2.01\,\% \ . \tag{3}$$

Knowing r_s, the effective penetration depth into the mirrors (L_p) can now be evaluated via [6]:

$$L_\mathrm{p} = \frac{\tanh[(N_\mathrm{g} - 1)r_\mathrm{s}]a}{r_\mathrm{s}} = 24.91\,\mathrm{\mu m} \ . \tag{4}$$

From these quantities, the effective resonator length $L_\mathrm{eff} = d + 2L_\mathrm{p}$ is found to be $L_\mathrm{eff} = 351.82\,\mathrm{\mu m}$. This quantity allows us to calculate the group velocity from the time domain data presented in the main text [Fig. 4c)]. Further, it is now possible to determine an effective quality factor of the resonator mirrors (Q_g) [5]:

$$Q_\mathrm{g} = \frac{\pi(d + 2L_\mathrm{p})}{\lambda_\mathrm{SAW}[1 - \tanh(r_\mathrm{s}N_\mathrm{g})]} \ . \tag{5}$$

Here, λ_p denotes the acoustic wavelength.

Due to the external quality factor being significantly larger than the internal quality factor Q_i, the total Q factor is dominated by Q_i, which can be separated into contributions from losses in the mirrors and propagation losses [5]:

$$\frac{1}{Q_\mathrm{i}} = \frac{1}{Q_\mathrm{g}} + \frac{v_\mathrm{gSAW}}{\pi \cdot f \cdot l_\mathrm{SAW}} \ . \tag{6}$$

In this equation f denotes the frequency of the supported mode and l_p denotes the SAW decay length. It is now possible to calculate the decay length from the previously established parameters: $l_\mathrm{p} = 730.47\,\mathrm{\mu m}$, which agrees well with the one found by evaluating the time domain data as seen in Supplementary Note 1D ($757\,\mathrm{\mu m}$).

D: Resonator in time domain

Following the approach detailed in the main text methods section, the frequency domain data of the resonator can be transformed into the time domain. Here, we demonstrate how information extracted from this time-domain transformation can be used to further characterize the resonator properties. Therefore, Supplementary Fig. 3 displays the off-resonant blue line cut presented in Fig. 4f) of the main text. Each peak visible corresponds to the SAW passing the IDT which is located in the center of the resonator. Knowing the effective resonator length L_{eff}, we can now calculate the SAW group velocity and the decay length. These values can then be compared to those obtained through other evaluation methods.

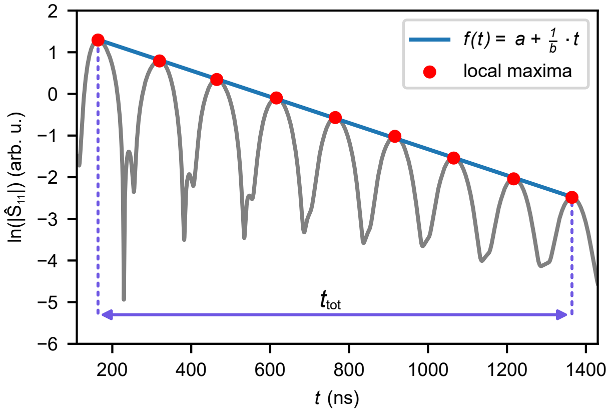

Supplementary Fig. 3: Natural logarithm of the time domain $|\hat{S}_{11}|$ reflection parameter in dependence of time t. The local maxima marked by the red points identify the single passes of the SAW through the resonator. By fitting a linear function to these maxima (blue line) the exponential decay $(1/b)$ is accessed. Further, t_{tot} marks the time the SAW takes to pass the resonator 8 times.

To better visualize the exponential nature of the SAW decay, the natural logarithm of the $|\hat{S}_{11}|$ data is shown in Supplementary Fig. 3. To find the decay length, the local maxima (red dots) are fitted using the linear function $f(t) = a + (1/b) \cdot t$, where a is the y-axis offset and $1/b$ represents the exponential decay constant. Knowing the SAW group velocity presented in Note 1B ($v_{\text{BLS}} = 2382\,\text{m}\,\text{s}^{-1}$), the decay length is calculated via $l_{\text{SAW}} = b \cdot v_{\text{BLS}} = 757\,\mu\text{m}$. This value agrees well with the previously calculated decay length of $730\,\mu\text{m}$.

In Supplementary Fig. 3, t_{tot} denotes the total time taken for the SAW to complete eight passes through the resonator. The group velocity is now calculated via $v_{\text{gSAW}} = \frac{8 \cdot L_{\text{eff}}}{t_{\text{tot}}} = 2344\,\text{m}\,\text{s}^{-1}$, which is again in agreement with the directly accessed group velocity employing timeresolved μBLS spectroscopy of $2382\,\text{m}\,\text{s}^{-1}$.

The strong consistency between the results obtained from various, distinct evaluation methods presented in Supplementary Note 1 underscores a comprehensive understanding of our SAW resonator's behavior.

Supplementary Note 2: Broad band FMR and VSM measurements

To find the saturation magnetization M_{s}, a vibrating sample magnetometer measurement was conducted on a reference YIG chip from the same wafer, utilizing the Physical Property Measurement System (PPMS) by Quantum Design. A hysteresis loop at room temperature was recorded, resulting in a saturation magnetization of $M_{\text{s}} = 127\,\text{kA}\,\text{m}^{-1}$, which is in reasonable agreement with literature values around $140\,\text{kA}\,\text{m}^{-1}$. Broadband ferromagnetic resonance spectroscopy was subsequently performed to find the crystal anisotropy field M_{ani} and the g-factor. However, for YIG films that are no longer thin films, many PSSW modes arise very close to the FMR frequency. This can be seen in Supplementary Fig. 4a), where the magnetic field derivative of the real part of the transmission parameter S_{21} is color-coded as a function of frequency and magnetic field [7]. From the lowest frequency signal visible as a function of field, the resonance positions are extracted and subsequently fitted to the Kittel equation [8]:

$$f = \frac{g\mu_{\text{B}}}{h}\sqrt{(\mu_0 H_{\text{res}} + \mu_0 H_{\text{ani}}) \cdot (\mu_0 H_{\text{res}} + \mu_0 H_{\text{ani}} + \mu_0 M_{\text{s}})}\ , \tag{7}$$

where f denotes the frequency, H_{res} the found resonance field, and h Planck's constant. The found g-factor is $g = 2.18$, and the anisotropy field is small, as expected: $\mu_0 H_{\text{ani}} = 0.99\,\text{mT}$. Due to the presence of numerous

other magnetic excitations near the FMR frequency, the extracted g-factor and anisotropy field should be considered approximate. A linecut at a fixed magnetic field is shown in Supplementary Fig. 4c), where clearly many magnetic excitations are visible. These excitations are compared to the first 10 calculated PSSW modes with an in-plane k-number of 0, as depicted on top of the measurement data in Supplementary Fig. 4b). The calculations agree well with the measured excitations and show that this behavior is predicted.

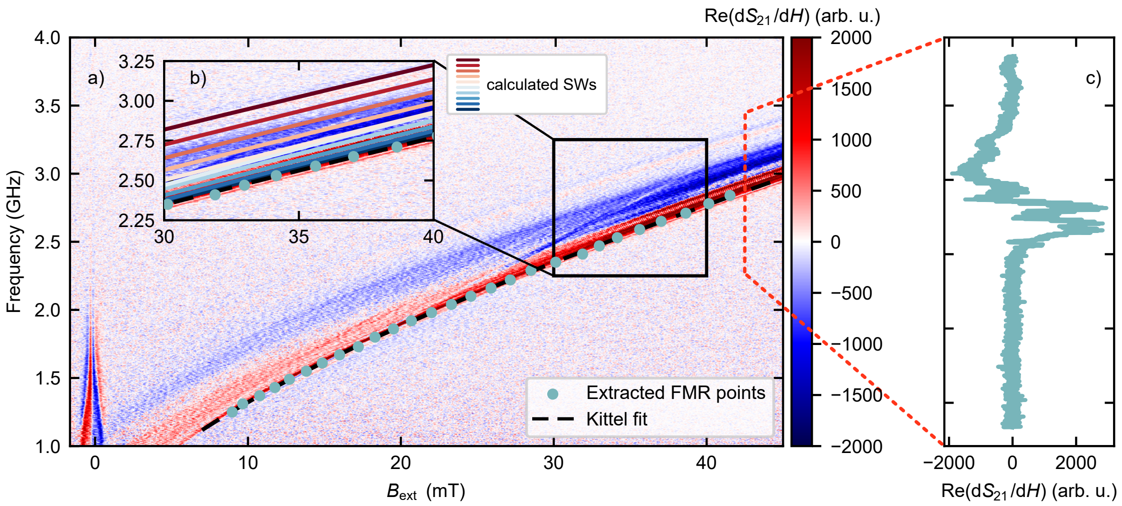

Supplementary Fig. 4: a) Broadband FMR measurement of YIG/ZnO sample. The lowest frequency mode exhibiting a clear field dependence represents the FMR and is used to characterize resonance positions. Kittel fit is shown as a black dashed line. b) First 10 calculated SW modes with an in-plane k-number of 0, depicted on top of the measurement data. Calculated modes agree well with the measured excitations. c) Signal at a fixed magnetic field showing a multitude of magnetic excitations.

Supplementary Note 3: Direct SW excitation and SW loss rate evaluation

As discussed in main text Sec. 2.1 , the spin wave (SW) loss rate is extracted from the direct SW excitation located in the center of the anticrossing. This direct excitation is attributed to a spurious RF current present in the microstructures [9]. A depiction of the central high-Q mode at 0° is shown in Supplementary Fig. 5a). The direct excitation is visible as the line centered in the anticrossing. This direct excitation also exhibits a smaller anticrossing feature, highlighted by the red dashed ellipse. To obtain the SW loss rate κ_m, the frequency linewidth of the direct excitation is extracted by fitting a Lorentzian to the linecut shown by the dashed red line. The fit and cut are shown in Supplementary Fig. 5b). Here, we find a loss rate of $\kappa_\mathrm{m}/2\pi = (1.24 \pm 0.02)\,\mathrm{MHz}$. To determine κ_m for other angles, we employed the approach outlined in Supplementary Ref. [10]. This method utilizes a simplified version of the Kalinikos-Slavin spin wave dispersion relation, from which the following expression for the angular frequency linewidth ($\Delta\omega$) is derived:

$$\Delta\omega = \alpha\sqrt{\left(\frac{1}{2}\gamma\mu_0(H_\mathrm{x}^\mathrm{dip} - H_\mathrm{y}^\mathrm{dip})\right)^2 + \omega^2}, \tag{8}$$

where ω is the angular frequency of the SW mode. The contributing fields $H_\mathrm{x}^\mathrm{dip}$ and $H_\mathrm{y}^\mathrm{dip}$ are given by:

$$H_\mathrm{x}^\mathrm{dip} = M_\mathrm{s}\frac{1-e^{-kd}}{kd}, \quad H_y^\mathrm{dip} = M_\mathrm{s}\left(1 - \frac{1-e^{-kd}}{kd}\right)\sin^2\phi\ . \tag{9}$$

In these Supplementary equations, M_s denotes the saturation magnetization, k the in-plane wavenumber, d the film thickness, ϕ the angle between the external field and the magnetization direction, and α denotes the Gilbert damping.

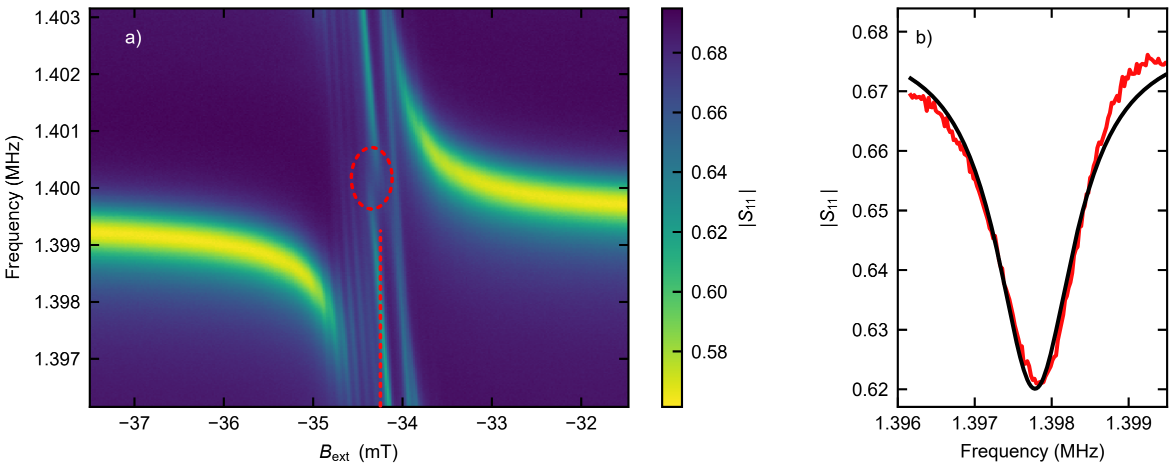

Supplementary Fig. 5: a) Anticrossing with fundamental SW excitation at 0° of central high-Q mode. Central line corresponds to direct SW excitation, exhibiting a secondary anticrossing (red dashed ellipse). Dashed red line marks the linecut used to evaluate the loss rate. b) Red line depicts the extracted linecut from a). It is fitted with a Lorentzian function (black) to extract the SW loss rate.

At the fixed angle of 0°, where all parameters in Supplementary Eq. (8) are known except for α, we solved for α, yielding $\alpha = 9 \times 10^{-4}$, which is in the right order of magnitude for YIG.

Additional magnetic-field–dependent spectral features are observed in the anticrossing region. The upper edge of the polaron branch associated with the high-Q mode at lower frequency appears at a slightly higher field than the direct SW excitation. When the field is decreased in this region, several narrow spectral lines emerge, which shift systematically with magnetic field.

The exact origin of these features is unclear. They cannot be attributed to PSSWs with the same in-plane wavevector as the SAW, as verified by the calculated PSSW dispersions shown in Fig. 2e) of the main text. One possibility is that these signals originate from weakly excited SW modes with different wavevectors, potentially driven by different stray magnetic fields generated by the microstructure. Alternatively, they may arise from modified boundary conditions at the YIG/ZnO interface, possibly due to surface roughness or defects introduced during ZnO sputtering, which can alter the magnetic pinning.

However, these features are low in amplitude and do not display clear hybridization behavior or anticrossing with the SAW mode. Importantly, they do not affect the frequency-domain analysis of the main anticrossing features and were therefore excluded from the quantitative evaluation presented in this work.

Supplementary Note 4: Angle dependent SW velocity

Our system enables coupling to magnons with a non-zero group velocity. To determine this velocity, we calculated the derivative of the spin wave angular frequency ω with respect to the wave vector k, $\mathrm{d}\omega/\mathrm{d}k$, derived from the general Kalinikos-Slavin dispersion relation. For each measured angle, this dispersion relation was evaluated at a fixed magnetic field corresponding to the observed anticrossing (B_{ac}). The spin wave group velocity (v_{gSW}) was then obtained by evaluating this derivative at the wave vector of the SAW. The calculated velocities are presented in Supplementary Fig. 6, which clearly shows that the maximum coupling observed at 0°, for instance, occurs at an angle where the SW group velocity is indeed non-zero.

Knowing the group velocity and the decay rate of the SWs, it is possible to calculate the maximum decay length $l_{\mathrm{SW}} = \frac{v_{\mathrm{gSW}}}{\kappa_{\mathrm{m}}/2\pi} \approx 500\,\mu\mathrm{m}$.

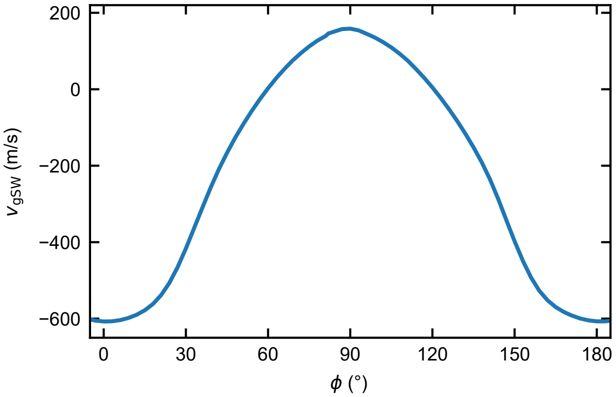

Supplementary Fig. 6: Spin wave group velocity v_{gSW} as a function of magnetic field direction ϕ.

Supplementary Note 5: Angle dependent coupling strength of all SW evaluated modes

Complementing the $n = 0$ mode presented in main text Fig. 3g), Supplementary Fig. 7 displays the coupling strength as a function of the angle ϕ (between the external magnetic field and the SAW wave vector) for all evaluated spin wave modes. The coupling strength g exhibits distinct qualitative and quantitative angular dependencies for different SW modes. This behavior is attributed to the varying degree of mode overlap between the spin wave and surface acoustic wave profiles as the angle changes.

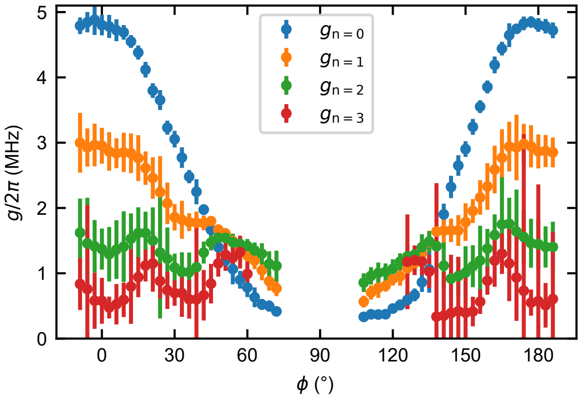

Supplementary Fig. 7: Coupling strength of the evaluated SW modes with respect to external field direction ϕ.

Supplementary Note 6: Simple model of Rabi-like oscillations

As discussed in main text Sec. 2.3, the Rabi frequency of the coupled magnon-phonon system can be calculated from the detuning δ and the coupling strength g, both accessible through frequency domain analysis. Following the standard approach for two-level systems [11], the probability $P_{\mathrm{m}}(t)$ of finding the system in the uncoupled magnon eigenstate at time t is given by Rabi's formula:

$$P_{\mathrm{m}}(t) = \frac{4g^2}{\omega_{\mathrm{rabi}}^2} \sin^2\left(\frac{\omega_{\mathrm{rabi}}}{2} t\right) \ , \tag{10}$$

where ω_{rabi} is the angular Rabi frequency, defined as:

$$\omega_{\mathrm{rabi}} = \sqrt{4g^2 + \delta^2} \ , \text{with} \quad \delta = \gamma |B_{\mathrm{ac}} - B_{\mathrm{ext}}| \ . \tag{11}$$

Here, the δ is determined by the linear shift of the SW dispersion with the applied magnetic field in the vicinity of the anticrossing, as discussed in the main text. This field shift is quantified by the difference between the anticrossing field B_{ac} and the external field B_{ext}. The resulting theoretical calculation of $P_{\mathrm{m}}(t)$ as a function of time and magnetic field is presented in Supplementary Fig. 8. The characteristic hat-like shape, also observed in the time-domain experimental data, is clearly visible.

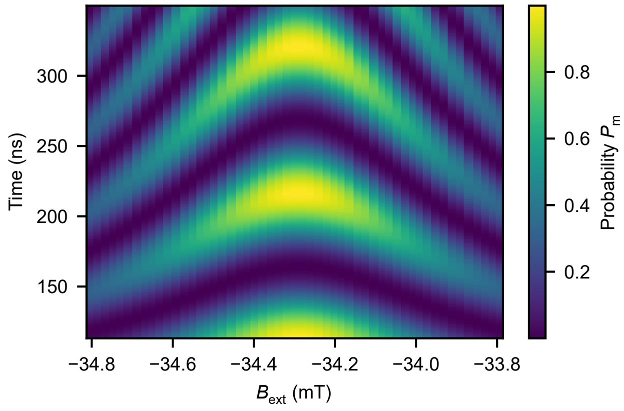

Supplementary Fig. 8: Rabi-like oscillations evaluated using the coupling strength g and detuning δ, accessed via frequency domain analysis. The characteristic hat-shaped probability to find the system in the magnon eigenstate $P_{\mathrm{m}}(t)$ is visible as a function of time and magnetic field (implied by the use of δ).

From the time-domain data shown in Fig. 4a) of the main text, we can further extract the coupling strength and decay rates of the system. To illustrate this, we analyze the two representative traces shown in main text Fig. 4c) in more detail. These traces are shown again in Supplementary Fig. 9. The blue curve corresponds to the uncoupled case, while the orange curve represents a coupled trace, chosen slightly off the exact anticrossing center.

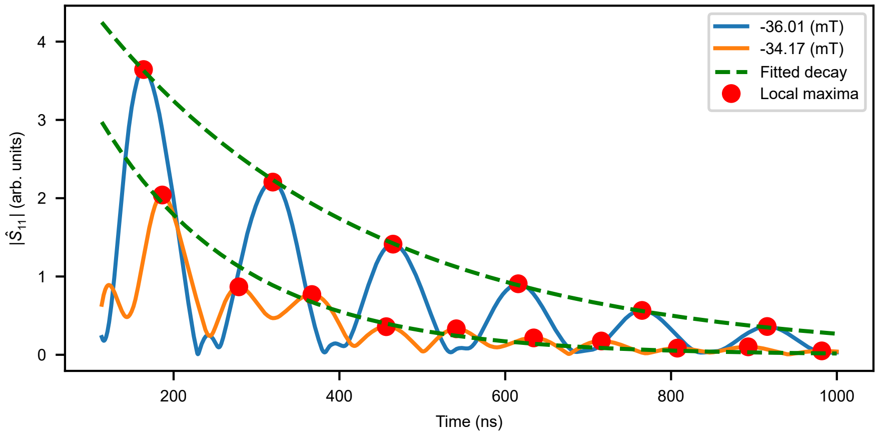

Supplementary Fig. 9: Time-domain representation of the $|\hat{S}_{11}|$ reflection parameter. The blue and orange curves correspond to the same line cuts shown in Fig. 4c) of the main text, representing the uncoupled SAW resonator (blue) and the coupled case (orange). Red dots indicate the local maxima used to fit exponential decay functions (green dashed lines) to determine the decay times. For the coupled case, the mean time interval between successive maxima provides the Rabi frequency.

We first determine the local maxima of the oscillations. For the coupled case (orange curve), the mean interval between successive maxima is $\bar{t}_{\mathrm{diff}} = (88.4 \pm 3.9)\,\mathrm{ns}$, corresponding to a Rabi frequency of $f_{\mathrm{rabi}} = (11.3 \pm 0.5)\,\mathrm{MHz}$. Using the detuning δ and rearranging Supplementary Eq. (11), we obtain a coupling strength $g/2\pi = (5.39 \pm 0.53)\,\mathrm{MHz}$ in agreement with the value extracted from the frequency-domain analysis. We then compare the decay rates derived from time-domain and frequency-domain data. Assuming exponential decay, we fit the amplitude of the local maxima of the oscillations to $A(t) = A_0 \cdot \exp\left(-2t\left(\frac{1}{\tau_{\mathrm{m}}} + \frac{1}{\tau_{\mathrm{p}}}\right)\right)$. From the uncoupled trace, we extract the SAW decay

time $\tau_\mathrm{p} = (642 \pm 6)$ ns. This value is then fixed when fitting the coupled case, yielding a magnon decay time $\tau_\mathrm{m} = (728 \pm 163)$ ns, corresponding to a decay rate $\kappa_\mathrm{m}/2\pi = (1.37 \pm 0.31)$ MHz. This value agrees well with the magnon decay rate determined from the direct SW excitation shown in Supplementary Note 3.

It is important to note that the simplified two-level model used here does not fully capture the complexity of our experimental system. In particular, it neglects the multiple concurrent SAW passes within the resonator, as well as the influence of spurious signals and the directly excited spin-wave mode observed at the anticrossing center (see Supplementary Note 3). Additional contributions, such as the polaron branch from a lower-frequency SAW mode, further reduce the quality of the time-domain data, leading to larger uncertainties in the extracted parameters. A more complete theoretical description, incorporating the full mode structure and accounting for the transient SAW resonator response, would be required to accurately model the time evolution of the strongly coupled system. Here, we employ the simplified model to demonstrate that decay rates and the coupling strength can be extracted consistently with the evaluation from the frequency domain, despite the complexities mentioned above.

Supplementary Note 7: Theoretical description of magnon-phonon coupling

This section discusses in detail the theoretical framework describing the magnon–phonon coupling strength in our device. The overall procedure entails an appropriate quantization of the magnetization and strain components resulting in their expression in terms of the magnon and phonon quasiparticles. This typical procedure allows us to obtain familiar expressions for coupled bosonic quasiparticles in terms of their ladder operators. We note two key features relevant to our device architecture. First, since we consider a system with low damping, we disregard the latter in our quantization procedure and reintroduce it at the end, as is typical in such considerations [12]. Second, our device architecture defines a magnetoacoustic cavity in a small fraction of the total inplane space available to the quasiparticles. This requires confinement of the quasiparticles of interest to this small inplane space and is intertwined with the effects of magnon-phonon coupling. In our analysis below, we assume that the magnons and phonons of interest are confined to the same physical inplane space thereby obtaining results that agree well with our experiments. The interplay between this confinement and magnon-phonon hybridization that allows us to make this assumption in the first place are then discussed further in Sec. 7 D.

Due to the YIG film being 1.98 µm thick, the numerical tool TetraX [13, 14] is used to access the SW mode profiles in this film. For the simulations, a saturation magnetization of $M_\mathrm{s} = 127\,\mathrm{kA\,m^{-1}}$ was used, as determined by vibrating sample magnetometry measurements on a reference chip. The exchange stiffness was set to $A = 3.75 \times 10^{-12}\,\mathrm{J\,m^{-1}}$. The geometry was modeled as a single-layer film with a thickness of 1980 nm and a uniform mesh spacing of 5 nm. Mode profiles were extracted at a wave vector of $k = 3.02\,\mathrm{rad\,\mu m^{-1}}$, corresponding to the SAW wave vector in the resonator discussed in Supplementary Note 1B.

From previous work it is known, that the initially excited SAW modes in a similar material stack are Rayleigh-like surface acoustic waves [2], however they have a nonvanishing shear-displacement component which can be amplified by the SAW resonator. We therefore allow all displacement components to enter the calculation. These elastic displacements are assumed to decay exponentially from the surface. This classical description of elastic and magnetization dynamics is then quantized and expressed in terms of quasiparticles- phonons and magnons. Employing the ensuing relations, magnetoelastic energy is expressed in terms of a Hamiltonian involving phonons and magnons, which directly yields the magnon-phonon coupling strength. We largely follow the approach in Supplementary Ref. [15] correcting and generalizing it as per the requirements of our system.

A: Outline of the quantization procedure

We introduce the relation between classical and quantum formalisms using the simple example of elastic displacements on a 1D string [16]. Consider the classical Hamiltonian:

$$H_\mathrm{1D} = \int_L \mathrm{d}x \, \left[\frac{1}{2}\rho \dot{u}^2 + \frac{1}{2} T \left(\frac{\partial u}{\partial x} \right)^2 \right] \tag{12}$$

describing a one-dimensional elastic displacement u on a string. Here, ρ is the mass density, T parametrizes the elastic potential energy, and L is the string length. In order to obtain the eigenmode profiles and frequencies, one typically expresses the displacement in terms of orthonormal functions $\phi_k(x)$ that satisfy the boundary conditions imposed on

the system:

$$u(x) = \sum_k q_k \phi_k(x), \tag{13}$$

where q_k parametrize the different contributions and k labels the distinct orthonormal functions which satisfy the orthonormality condition:

$$\int_L dx\ \phi_k(x)\phi^*_{k'}(x) = \delta_{k,k'}. \tag{14}$$

With these substitutions, one can proceed towards bringing the classical Hamiltonian [Supplementary Eq. (12)] into a diagonal form in terms of the different k modes. From this point on, one can quantize by identifying the canonical position and momentum, making them operators, and postulating the appropriate commutation relation between them. The procedure is described, for example, in Supplementary Ref. [16].

We pause to discuss the key points in this mathematical procedure. First, the orthonormal functions $\phi_k(x)$ that satisfy Supplementary Eq. (14) become the quasiparticle wavefunctions after the quantization procedure. Thus, within the quantum framework, their orthonormality ensures that the different quasiparticles do not interact (disregarding higher order nonlinearities) and that the probability of finding a single quasiparticle somewhere in all space is unity. Secondly, the commonly employed example of an infinite medium [16] is modeled via the periodic boundary conditions: $u(x + L) = u(x)$ which results in plane waves basis $\phi_k(x) = (1/\sqrt{L})\ \exp(ikx)$ with discrete values of k imposed by the condition: $u(x+L) = u(x)$. In this manner, the boundary conditions are the key to determining the orthonormal basis functions irrespective of whether we use classical or quantum description. If one considered a finite medium and some confinement boundary conditions on the edges, the exact nature of the basis functions $\phi_k(x)$ depends on the precise boundary conditions.

These two points assume an especially important significance in our investigated devices. Since we are considering two coupled fields, magnetization and strain, with coupled boundary conditions [17, 18], we should ideally determine the corresponding orthonormal wavefunctions for the magnetoelastic modes self-consistently with the boundary conditions. This appears to be infeasible in our system since the predominantly elastic boundary conditions that define our acoustic cavity and separate it from the remaining part of the large sample are not easily accounted for, even numerically. Thus, we take a simplified approach in our theoretical analysis and assume that the magnons and phonons are limited to the defined acoustic cavity. We consider the good agreement with experiments as a posterior justification of this assumption and further examine it from a theory standpoint in Sec. 7 D.

B: Quantization of the phonon and magnon modes

We first consider the elastic degrees of freedom with a coordinate system such that the wave vector is along x axis and the film out-of-plane direction is along the z axis. Furthermore, we consider the three-dimensional elastic displacement with components $u_x\hat{x}$, $u_y\hat{y}$, and $u_z\hat{z}$. We consider a SAW which is confined by an acoustic resonator such that it forms a standing wave profile in the x direction and decays exponentially along z axis with the surface lying at $z = 0$. With these conditions and following the quantization procedure outlined in Sec. 7 A, we may write the quantized expressions for the displacements:

$$\tilde{u}_x = \sqrt{\frac{\hbar}{\rho A \omega_p}} t_x f(z) \sin(k_p x + \phi_p)(\tilde{a} + \tilde{a}^\dagger), \tag{15}$$

$$\tilde{u}_y = \sqrt{\frac{\hbar}{\rho A \omega_p}} t_y f(z) \sin(k_p x + \phi_p)(\tilde{a} + \tilde{a}^\dagger), \tag{16}$$

$$\tilde{u}_z = \sqrt{\frac{\hbar}{\rho A \omega_p}} t_z f(z) \sin(k_p x + \phi_p)(\tilde{a} + \tilde{a}^\dagger), \tag{17}$$

where ρ is the material density assumed to be spatially uniform for simplicity, A is the area of confinement due to the acoustic resonator, ω_p and k_p are the angular frequency and wavenumber of the phonon mode under consideration, $\tilde{a}$ is the annihilation operator for the phonon mode, $t_{x,y,z}$ parametrize the relative strength of the displacement components and they follow the constraint $t_x^2 + t_y^2 + t_z^2 = 1$, and $f(z)$ parametrizes the spatial dependence and decay of the mode. Here, ϕ_p is the standing wave phase that depends on the precise boundary conditions. We note and emphasize that the above quantization assumes that the phonon modes are fully confined to the cavity with an area A, which is a

small fraction of the total area available in our extended devices. Due to the complex nature of our acoustic cavity, we are unable to fully ascertain ϕ_{p} and assume it to be zero. We discuss this issue further below. In our simplified model, we assume $f(z)$ to be a simple exponential decay that follows the normalization condition:

$$\int_0^\infty dz|f(z)|^2 = 1, \tag{18}$$

$$\implies f(z) = \sqrt{\frac{2}{z_{\mathrm{d}}}} e^{-\frac{z}{z_{\mathrm{d}}}}, \tag{19}$$

where z_{d} parametrizes the decay length. Employing Supplementary Eqs. (15) - (17) with $\phi_{\mathrm{p}} = 0$, we obtain the expression for the relevant components of the strain tensor:

$$\tilde{e}_{\mathrm{xx}} = \frac{\partial \tilde{u}_{\mathrm{x}}}{\partial x} = k_{\mathrm{p}} \sqrt{\frac{\hbar}{\rho A \omega_{\mathrm{p}}}} t_{\mathrm{x}} f(z) \cos(k_{\mathrm{p}} x)(\tilde{a} + \tilde{a}^\dagger), \tag{20}$$

$$\tilde{e}_{\mathrm{xz}} = \frac{1}{2}\left(\frac{\partial \tilde{u}_{\mathrm{x}}}{\partial z} + \frac{\partial \tilde{u}_{\mathrm{z}}}{\partial x}\right), \tag{21}$$

$$= \frac{1}{2}\sqrt{\frac{\hbar}{\rho A \omega_{\mathrm{p}}}} t_{\mathrm{x}} f'(z) \sin(k_{\mathrm{p}} x)(\tilde{a} + \tilde{a}^\dagger)$$

$$+ \frac{k_{\mathrm{p}}}{2}\sqrt{\frac{\hbar}{\rho A \omega_{\mathrm{p}}}} t_{\mathrm{z}} f(z) \cos(k_{\mathrm{p}} x)(\tilde{a} + \tilde{a}^\dagger). \tag{22}$$

$$\tilde{e}_{\mathrm{xy}} = \frac{1}{2}\left(\frac{\partial \tilde{u}_{\mathrm{x}}}{\partial y} + \frac{\partial \tilde{u}_{\mathrm{y}}}{\partial x}\right), \tag{23}$$

$$= \frac{k_{\mathrm{p}}}{2}\sqrt{\frac{\hbar}{\rho A \omega_{\mathrm{p}}}} t_{\mathrm{y}} f(z) \cos(k_{\mathrm{p}} x)(\tilde{a} + \tilde{a}^\dagger), \tag{24}$$

$$\tilde{e}_{\mathrm{yz}} = \frac{1}{2}\left(\frac{\partial \tilde{u}_{\mathrm{y}}}{\partial z} + \frac{\partial \tilde{u}_{\mathrm{z}}}{\partial y}\right), \tag{25}$$

$$= \frac{1}{2}\sqrt{\frac{\hbar}{\rho A \omega_{\mathrm{p}}}} t_{\mathrm{y}} f'(z) \sin(k_{\mathrm{p}} x)(\tilde{a} + \tilde{a}^\dagger) \tag{26}$$

$$\tilde{e}_{\mathrm{zz}} = \frac{\partial \tilde{u}_{\mathrm{z}}}{\partial z} = \sqrt{\frac{\hbar}{\rho A \omega_{\mathrm{p}}}} t_{\mathrm{z}} f'(z) \sin(k_{\mathrm{p}} x)(\tilde{a} + \tilde{a}^\dagger). \tag{27}$$

The corresponding quantized expressions for the magnetization components in a coordinate system (see Supplementary Fig. 10) with z' axis aligned with the equilibrium magnetization are obtained as [15, 19]:

$$\tilde{m}_{\mathrm{x}'} = \sqrt{\frac{\gamma \hbar M_{\mathrm{s}}}{A}} \cos(k_{\mathrm{p}} x + \phi_{\mathrm{m}}) \mathcal{M}_{\mathrm{ip}}(z) \left(\tilde{c} + \tilde{c}^\dagger\right), \tag{28}$$

$$\tilde{m}_{\mathrm{y}'} = \frac{1}{i}\sqrt{\frac{\gamma \hbar M_{\mathrm{s}}}{A}} \cos(k_{\mathrm{p}} x + \phi_{\mathrm{m}}) \mathcal{M}_{\mathrm{oop}}(z) \left(\tilde{c}^\dagger - \tilde{c}\right), \tag{29}$$

where M_{s} is the saturation magnetization, γ is the gyromagnetic ratio magnitude, $\tilde{c}$ is the magnon annihilation operator, $\mathcal{M}_{\mathrm{ip,oop}}(z)$ capture the spatial profile and ellipticity of the magnetostatic mode which is frequency and wavenumber matched with the phonon mode. Please note our assumption in the quantization above that the magnon modes are confined to the acoustic cavity occupying an area A, which is a small fraction of the total area over which the magnetic film extends. The spatial profile of the magnetostatic mode is evaluated numerically within the Landau-Lishitz framework and is considered an input to our analysis. Further, it obeys the normalization condition

$$\int_{FM} \mathrm{d}z \; \mathcal{M}_{\mathrm{ip}}(z) \mathcal{M}^*_{\mathrm{oop}}(z) = 1, \tag{30}$$

where the integration is over the ferromagnet (FM) thickness. When $\mathcal{M}_{\mathrm{ip,oop}}$ are available only for propagating modes, one should obtain the effective $\mathcal{M}_{\mathrm{ip,oop}}$ by averaging over the forward and backward propagating modes, and then normalize the resulting function using the Supplementary equation above. A more detailed quantization procedure can be found in Ref. [20, 21].

As in the case of phonons, $\phi_{\rm m}$ depends on the exact boundary conditions for the magnetization. There is an extra layer of complication here. One might expect the magnons to not be confined by the acoustic resonator at all. However, in the regime of interest to us, phonons and magnons hybridize and the acoustic boundary conditions affect and confine the hybrid quasiparticle. Taking this implicitly into account, in our considerations above, we have already assumed that the magnon is confined to the region defined by the acoustic resonator. This is yet another reason for our inability to rigorously account for the boundary conditions experienced by the hybrid quasiparticle. We again assume $\phi_{\rm m} = 0 = \phi_{\rm p}$ and that any error introduced by these assumptions does not affect the qualitative physics and dependencies.

C: Magnetoelastic coupling to magnon-phonon coupling

We are now ready to consider the magnetoelastic energy, expressed classically in a cubic [100] system as

$$H_{\rm mec} = \int_{FM} {\rm d}^3r \; b_1 \left(\frac{m_{\rm x}^2}{M_{\rm s}^2} e_{\rm xx} + \frac{m_{\rm z}^2}{M_{\rm s}^2} e_{\rm zz} \right) + 2b_2 \left(\frac{m_{\rm x} m_{\rm z}}{M_{\rm s}^2} e_{\rm xz} + \frac{m_{\rm x} m_{\rm y}}{M_{\rm s}^2} e_{\rm xy} + \frac{m_{\rm y} m_{\rm z}}{M_{\rm s}^2} e_{\rm yz} \right), \tag{31}$$

where we have disregarded the term containing the vanishing strain component e_{yy}. Owing to the weak anisotropies in YIG and to maintain clarity and brevity in the theoretical treatment, the coordinate system used to express the magnetoelastic energy is not rotated into the [111] crystallographic frame of the employed YIG film. The magnetoelastic constants are denoted by $b_1 = 3.48 \times 10^5\,{\rm J\,m^{-3}}$ and $b_2 = 3.48 \times 10^5\,{\rm J\,m^{-3}}$ [22]. Assuming that an in-plane applied magnetic field saturates the equilibrium magnetic moment along $\hat{z}'$ (Supplementary Fig. 10), we obtain the following relation between the magnetization in the two coordinate frames:

$$m_{\rm x} = M_{\rm s} \cos\phi - m_{\rm x'} \sin\phi, \quad m_{\rm z} = m_{\rm y'}, \quad m_{\rm y} = M_{\rm s} \sin\phi + m_{\rm x'} \cos\phi, \tag{32}$$

where $m_{\rm x'}$ and $m_{\rm y'}$ are the small deviations characterizing the magnetization dynamics.

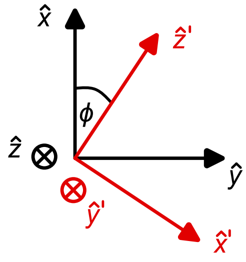

Supplementary Fig. 10: Schematic for the two coordinate systems. The primed coordinate system is used to characterize the magnetic ground state (magnetization pointing along $\hat{z}'$) and dynamics, while the unprimed coordinate system describes the lattice better. ϕ is thus the angle between the equilibrium magnetic moment and the wave vector direction $\hat{x}$.

Employing the transformations above in Supplementary Eq. (31) and retaining only the terms linear in the small variables $m_{x'}$ and $m_{y'}$, we obtain

$$H_{\rm mec} = \int_{FM} {\rm d}^3r \; 2b_2 \left(\cos\phi \frac{m_{\rm y'}}{M_{\rm s}} e_{\rm xz} + \cos(2\phi) \frac{m_{\rm x'}}{M_{\rm s}} e_{\rm xy} + \sin\phi \frac{m_{\rm y'}}{M_{\rm s}} e_{\rm yz} \right) - b_1 \sin(2\phi) \frac{m_{\rm x'}}{M_{\rm s}} e_{\rm xx}. \tag{33}$$

Employing Supplementary Eqs. (20)-(29) in Supplementary Eq. (33) above and making the rotating wave approximation (i.e., disregarding terms of the sort $\tilde{a}\tilde{c}$ and $\tilde{a}^\dagger\tilde{c}^\dagger$), we obtain the quantum Hamiltonian for magnon-phonon coupling:

$$\tilde{H}_{\rm mec} = -\hbar g_1 \left(\tilde{a}\tilde{c}^\dagger + \tilde{a}^\dagger\tilde{c} \right) - \hbar i g_2 \left(\tilde{a}\tilde{c}^\dagger - \tilde{a}^\dagger\tilde{c} \right), \tag{34}$$

where

$$g_1 = \frac{k_{\rm p}}{2} \sqrt{\frac{\gamma}{\rho \omega_{\rm p} M_{\rm s}}} \, \left[b_1 \, I_{\rm x,ip}(\phi) \, \sin(2\phi) - b_2 \, I_{\rm y,ip}(\phi) \, \cos(2\phi) \right], \tag{35}$$

$$I_{\rm x,ip}(\phi) = \int_{FM} dz \; t_{\rm x} f(z) \mathcal{M}_{\rm ip}(z), \tag{36}$$

$$I_{\rm y,ip}(\phi) = \int_{FM} dz \; t_{\rm y} f(z) \mathcal{M}_{\rm ip}(z), \tag{37}$$

$$g_2 = \frac{k_{\rm p}}{2} \sqrt{\frac{\gamma}{\rho \omega_{\rm p} M_{\rm s}}} \, b_2 \, I_{\rm z,oop}(\phi) \, \cos(\phi), \tag{38}$$

$$I_{\rm z,oop}(\phi) = \int_{FM} dz \; t_{\rm z} f(z) \mathcal{M}_{\rm oop}(z). \tag{39}$$

With these definitions, Supplementary Eq. (34) can be expressed as:

$$\tilde{H}_{\rm mec} = -\hbar \left(g \; \tilde{a} \tilde{c}^{\dagger} + g^{*} \; \tilde{a}^{\dagger} \tilde{c} \right),$$
$$g = g_1 + i g_2. \tag{40}$$

The ensuing gap in the anticrossing is $\hbar 2|g| = \hbar 2 \sqrt{g_1^2 + g_2^2}$. Further, we may now account for the nonzero dissipation of the uncoupled magnon and phonon modes by allowing their frequencies to be complex with the imaginary part capturing the dissipation [12] resulting in our employed Eq. (1) in the main text.

Supplementary equations (35) - (40) constitute our final result and we pause to discuss their physical significance. The ϕ dependence of g_1 [g_2] is governed by $I_{\rm x,ip}(\phi)$ and $I_{\rm y,ip}(\phi)$ [$I_{\rm z,oop}(\phi)$], which in turn depends on the ellipticity of the magnon mode and the spatial overlap between the phonon and magnon wavefunctions, and by the factors $\sin(2\phi)$ and $\cos(2\phi)$ [$\cos(\phi)$] coming directly from the form of magneto-elastic coupling, Supplementary Eq. (33). The relative importance of g_1 and g_2 is additionally controlled by the factors $t_{\rm x,y,z}$ which parametrize the relative magnitude of the different displacements in the phonon mode.

D: Interplay between confinement and magnon-phonon hybridization

In our considerations above, we have assumed the uncoupled magnon and phonon modes to be confined by the acoustic cavity to a small fraction of the total available inplane area in our bilayer device. As discussed above (Sec. 7 A), this is a simplified approach to capture the key physics of an otherwise theoretically intractable problem. We now examine this approach further.

Let us first consider how our results would change if we assume that the magnons are delocalized over an area A_m much larger than the acoustic cavity area A. That would require us to replace A by A_m in Supplementary Eqs. (28) and (29), which eventually would lead to the multiplication of our theoretically evaluated coupling strength [Supplementary Eq. (40)] by a factor $\sqrt{A/A_m}$. Considering A_m to be the total area of our large sample, this would make the theoretically evaluated magnon-phonon coupling to be vanishingly small thereby precluding our observed strong coupling. Thus, we find that a confinement of the magnon mode to the small area defined by the acoustic cavity is fundamentally important for our observation of the strong coupling and the consequent magnon-polaron formation.

In the above chain of arguments, one may consider that A_m could be estimated not by the total sample volume but by the square of magnon decay length. Such an accounting of damping is not feasible in this theoretical framework (Sec. 7 A) and could lead to misleading results. Furthermore, an explicit inclusion of damping considering a more advanced theoretical framework does not discount the fact that the magnetoelastic boundary conditions will impose reflection of a magnon-polaron by the acoustic cavity, as we now discuss.

Our actual device designs an acoustic cavity for a specific range of frequencies due to the series of electrical contacts deposited on the piezoelectric layer. This cavity and confinement provide rich physics by itself, which we do not attempt to capture. Instead, we try to appreciate the qualitative phenomena at play in our device by discussing a very different and simplified acoustic cavity formed by a film in vacuum. In our hypothetical acoustic cavity, the elastic waves propagate in the out-of-plane direction and are reflected by the film surfaces resulting in their confinement to the film. These reflections can be understood as arising from the fact that the energy carried by the elastic waves is not allowed to escape the film. In a mathematical rigorous theory [18], imposing of the energy flow continuity provides the boundary conditions, which for our hypothetical acoustic cavity will mean that specific stress and strain components need to be zero at the film surfaces. If we now consider the same film to be a magnetic material hosting

coupled magnetoelastic modes, the same energy continuity condition yields a new boundary condition that includes magnetization components in addition to the strain components [18]. This already provides the basic ingredient to how a purely acoustic cavity could confine a magnon mode by imposing magnetic boundary conditions. Furthermore, close to the anticrossing region in the dispersion diagram and assuming strong coupling, the energy current carried by the coupled magnetoelastic modes bears comparable contributions in the elastic and magnetic degrees of freedom [18]. Thus, the energy flow continuity across an interface already necessitates the reflection of a magnon-polaron without looking at the detailed procedure of boundary conditions. When the coupling is weak, magnons and phonons continue to exist as independent particles, no such reflection is necessary.

E: Exemplary coupling strengths calculated employing the model

The theoretical model described above was employed to calculate the coupling strength as a function of different combinations of the displacement weighting factors ($t_{x,y,z}$) and the SAW decay length (z_d). The magnetic mode profiles were obtained from TetraX [13, 14] simulations conducted using the experimentally determined anticrossing field (B_{ac}) and a fixed wave vector equal to that of the SAW. In Supplementary Fig. 11a), the three cases representing pure displacement components are shown. These cases represent scenarios where only one displacement component is considered ($t_i = 1$, with the other two set to zero). The resulting angular dependence of the coupling strength exhibits maxima at different angles depending on the dominant displacement component. Notably, the maximum coupling occurs at 0° only when the y-displacement component is dominant ($t_y = 1$). Panel b) illustrates the coupling strength calculated using the combination $t_y = 0.89$ and $t_z = 0.46$. With varying SAW decay length, the coupling strength changes in magnitude. If employing a less symmetric decay function for the SAW is assumed, instead of a purely exponential one, it allows for modifications to the symmetry of the coupling strength's angular dependence. Lastly, panel c) shows different combinations of $t_{x,y,z}$, changing the ratio between t_x and t_z. The parameters for the calculations are detailed in the figure caption. The specific parameters used for the theoretical curve presented in main text Fig. 3g) are $t_y = 0.89$, $t_z = 0.46$, $t_x = 0$ with a decay length of $z_d = 1.465\,\mu m$.

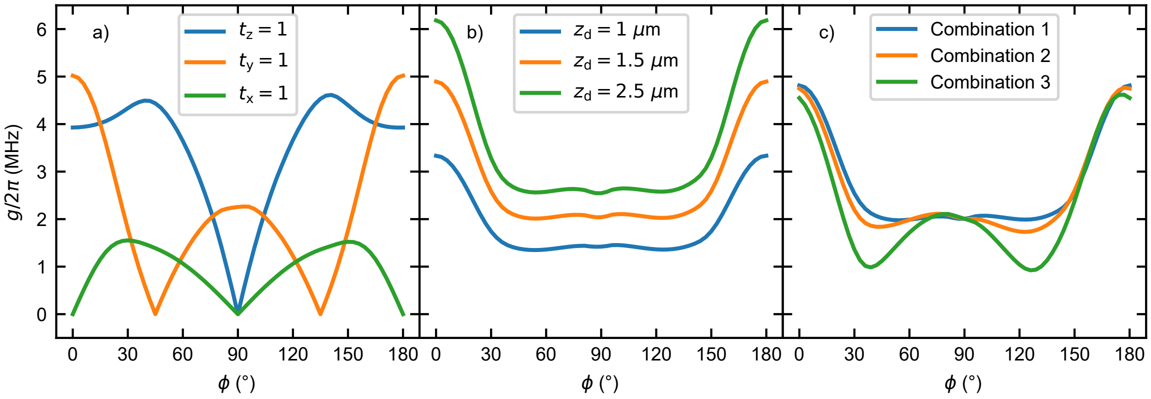

Supplementary Fig. 11: a) Theory curves for $z_d = 1.465\,\mu m$, where only displacements in one direction are taking into account. This is achieved by setting $t_{x,y,z}$ to one. b) Theory curves for $t_x = 0$, $t_y = 0.89$, $t_z = 0.46$ with varying z_d. c) Theory curves for $z_d = 1.465\,\mu m$, with varying displacement $u_{x,z}$ contributions. Combination 1: $t_x = 0$, $t_y = 0.89$, $t_z = 0.46$. Combination 2: $t_x = 0.2$, $t_y = 0.89$, $t_z = 0.41$. Combination 3: $t_x = 0.4$, $t_y = 0.89$, $t_z = 0.22$.

Supplementary Note 8: Sample fabrication

The device was fabricated on a 1.98 µm thick YIG layer grown on a 500 µm GGG substrate. A 976 nm thick piezoelectric ZnO layer was deposited onto the YIG via radio frequency magnetron sputtering, following a presputter cleaning of the YIG surface.

Microstructures were defined using a double-layer PMMA resist (PMMA 200K 4% AR-P 649.04, PMMA 950K 2% AR-P 679.02) patterned by electron beam lithography (EBL) at 20 kV. A conductive polymer (Espacer 300Z) was used to mitigate charging during EBL. The resist was developed in a 7:3 isopropanol to water solution. An oxygen plasma etch was used to remove residual PMMA in the exposed areas.

The fabrication process involved three EBL and lift-off steps using electron beam evaporation:

1. Alignment marks and a dose test were fabricated using a 5 nm Ti / 80 nm Au stack.
2. IDTs and SAW mirror structures were patterned using a 5 nm Ti / 30 nm Au stack in a single writing step for precise alignment.
3. Contact pads were fabricated using a 5 nm Ti / 150 nm Au stack with a thicker PMMA resist double layer (PMMA 600K 4% AR-P 669.04, PMMA 950K 2% AR-P 679.0) to ensure a sufficient undercut for lift-off.

The fabrication was performed at the nano structuring center at RPTU in Kaiserslautern, with ZnO sputtering carried out in the Clarendon Laboratory in Oxford.

Supplementary Note 9: Quality factor dependence on gated passes

Figure 4e) in the main text shows time-gated resonator signals after different numbers of gated passes with the gating procedure described in the method section. Here, we expand on the effect of time gating on the extracted Q factor of the resonator and comment on how this can be further exploited in experimental analysis.

By transforming the zero-field SAW resonator spectrum from the frequency to the time domain, distinct round-trips of the SAW pulse become visible, as shown in Supplementary Fig. 3 and detailed in Supplementary Note 1D. These individual passes can be selectively gated and converted back to the frequency domain for analysis. To illustrate the influence of both the number of included passes and the choice of window function, we present the extracted Q factors for various gating conditions in Supplementary Fig. 12.

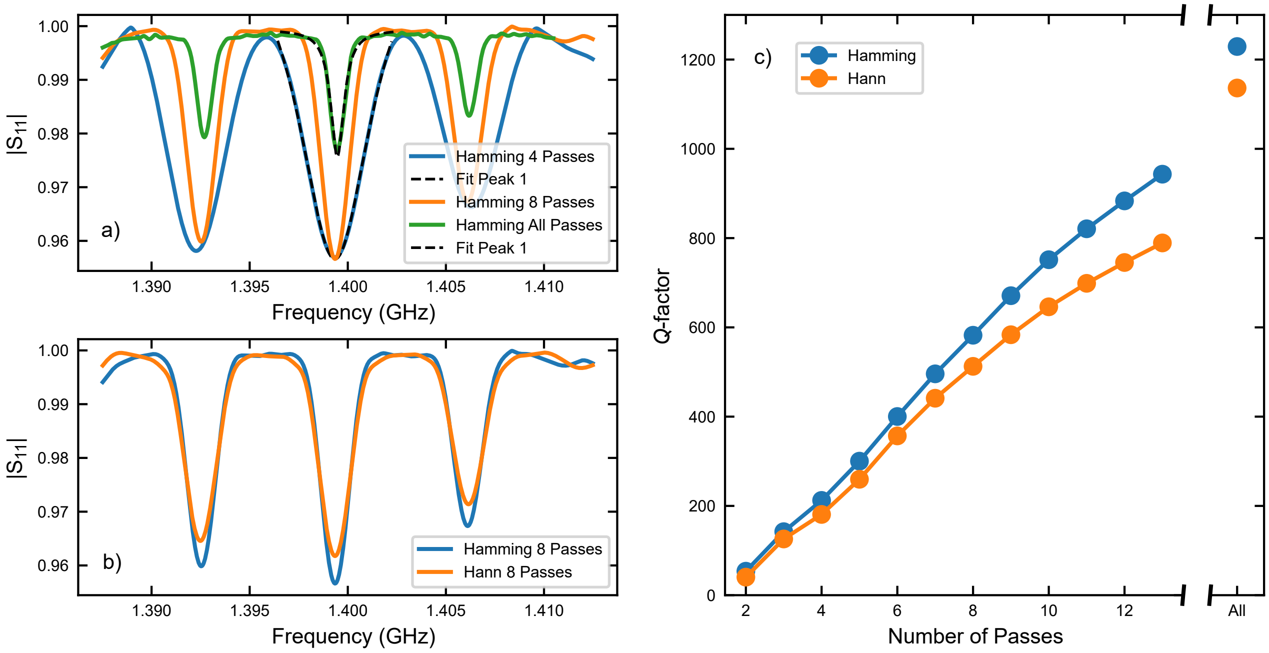

Supplementary Fig. 12: a) Time-domain gated spectra using a Hamming window including 4, 8, and all visible SAW passes. A clear trend of decreasing spectral bandwidth with increasing number of gated passes is observed, indicating an increase in the Q factor. Two representative fits to Eq. (3) of the main text are shown as dashed black lines. b) Comparison of the Hamming and Hann window functions applied for gating. c) Extracted Q factors as a function of the number of gated passes for each window function. With increasing pass count, the Q factor increases at a similar rate for both windows, with the maximum Q slightly exceeding the ungated case.

Supplementary Fig. 12a) shows how the SAW resonance bandwidth changes as the number of included passes in the gating window increases, using a Hamming window. Two representative fits to Eq. (3) of the main text are shown as dashed black lines. The corresponding extracted Q factors are plotted in panel c). Panel b) compares two commonly used window functions — Hann, and Hamming — applied to the same 8-pass gated data. Both the

Hann and Hamming windows are designed to suppress sidelobes in the Fourier transform that originate from sharp time-domain cutoffs. As expected, they produce similar spectral shapes, with only minor differences in dip depth.

Panel c) summarizes the dependence of the extracted Q factor on the number of gated passes for both window types. As expected, the Q factor increases with the number of passes, approaching and even slightly exceeding the value obtained from the ungated data in the main text. This improvement is attributed to the suppression of short-timescale noise contributions, such as direct electromagnetic crosstalk, which are effectively removed by time gating.

The observed increase in Q with longer time-domain windows is consistent with the expected behavior of the resonator: as the system evolves in time, the transient effects decay, and the later passes reflect the steady-state response, which is narrower in frequency.

To directly observe this behavior experimentally, several approaches are possible. One option is to use a VNA with a sufficiently large measurement bandwidth, where excitation and detection start simultaneously. In this case, individual frequency points can be sampled before the resonator reaches its steady state. By correlating the known timing of each SAW pass with the corresponding spectral content, the effective bandwidth can be adjusted accordingly.

Alternatively, a pulsed excitation scheme using a fast microwave switch combined with a real-time oscilloscope allows direct measurement of the time-resolved response of the resonator. This setup can capture the transient dynamics and the evolution of the SAW signal over multiple passes.

Finally, timeresolved microfocused Brillouin light scattering offers another powerful method to probe the SAW bandwidth inside the resonator. By controlling the pulse duration and frequency, and measuring at specific time delays corresponding to individual passes, the temporal, spectral and even spacial evolution of the acoustic modes can be observed.

Supplementary References

[1] Laidoudi, F., Caliendo, C., Kanouni, F. & Amara, S. Surface acoustic wave characteristics in zno thin films deposited on different silicon-based multilayered substrates. *Appl. Phys. A Mater. Sci. Process.* **131** (2025).

[2] Ryburn, F. *et al.* Generation of gigahertz-frequency surface acoustic waves in $Y_3Fe_5O_{12}$/ZnO heterostructures. *Phys. Rev. Appl.* **23**, 034062 (2025).

[3] Sebastian, T., Schultheiss, K., Obry, B., Hillebrands, B. & Schultheiss, H. Micro-focused brillouin light scattering: imaging spin waves at the nanoscale. *Front. Phys.* **3** (2015).

[4] Geilen, M. *et al.* Interference of co-propagating rayleigh and sezawa waves observed with micro-focused brillouin light scattering spectroscopy. *APL* **117**, 213501 (2020).

[5] Manenti, R. *et al.* Surface acoustic wave resonators in the quantum regime. *Phys. Rev. B* **93** (2016).

[6] Schuetz, M. J. A. *et al.* Universal quantum transducers based on surface acoustic waves. *Phys. Rev. X* **5**, 031031 (2015).

[7] Maier-Flaig, H. *et al.* Note: Derivative divide, a method for the analysis of broadband ferromagnetic resonance in the frequency domain. *Rev. Sci. Instrum.* **89** (2018).

[8] Kittel, C. On the theory of ferromagnetic resonance absorption. *Phys. Rev.* **73**, 155–161 (1948).

[9] Kunz, Y. *et al.* Efficient spin-wave excitation by surface acoustic waves in ultralow-damping yttrium iron garnet–zinc oxide heterostructures. *Phys. Rev. Appl.* **24** (2025).

[10] Weiler, M. Magnetization dynamics and spin torques in exchange–coupled spin systems (2019). Habilitation Thesis.

[11] Cohen-Tannoudji, C., Diu, B. & Laloë, F. *Quantum mechanics. Volume 1: Basic concepts, tools, and applications* (Wiley-VCH Verlag GmbH & Co. KGaA, Weinheim, 2020), second edition edn.

[12] Bai, L. *et al.* Control of the magnon–photon coupling. *IEEE Transactions on Magnetics* **52**, 1–7 (2016).

[13] Körber, L. *et al.* TetraX: Finite-Element Micromagnetic-Modeling Package (2022).

[14] Körber, L., Quasebarth, G., Otto, A. & Kákay, A. Finite-element dynamic-matrix approach for spin-wave dispersions in magnonic waveguides with arbitrary cross section. *AIP Adv.* **11**, 095006 (2021).

[15] Komiyama, H. *et al.* Quantitative evaluation method for magnetoelastic coupling between surface acoustic waves and spin waves using electrical and optical measurements (2024). Preprint on arXiv.

[16] Kittel, C. *Quantum Theory of Solids* (Wiley, 1963).

[17] Kamra, A. & Bauer, G. E. Actuation, propagation, and detection of transverse magnetoelastic waves in ferromagnets. *Solid State Commun.* **198**, 35–39 (2014).

[18] Kamra, A., Keshtgar, H., Yan, P. & Bauer, G. E. W. Coherent elastic excitation of spin waves. *Phys. Rev. B* **91**, 104409 (2015).

[19] Kamra, A. & Belzig, W. Magnon-mediated spin current noise in ferromagnet | nonmagnetic conductor hybrids. *Phys. Rev. B* **94**, 014419 (2016).

[20] Mills, D. Quantum theory of spin waves in finite samples. *J. Magn. Magn. Mater* **306**, 16–23 (2006).

[21] Zhang, X., Bauer, G. E. & Yu, T. Unidirectional pumping of phonons by magnetization dynamics. *Phys. Rev. Lett.* **125**, 077203 (2020).

[22] Comstock, R. Magnetoelastic coupling constants of the ferrites and garnets. *Proc. IEEE* **53**, 1508–1517 (1965).
